# Autophagy‐related genes polymorphism in hepatitis B virus‐associated hepatocellular carcinoma: A systematic review

**DOI:** 10.1002/iid3.1182

**Published:** 2024-02-14

**Authors:** Parastoo Yousefi, Alireza Tabibzadeh, Abdulhussain Kadhim Jawaziri, Mohsen Mehrjoo, Mandana Akhavan, Leila Allahqoli, Hamid Salehiniya

**Affiliations:** ^1^ Department of Virology, School of Medicine Iran University of Medical Sciences Tehran Iran; ^2^ Department of Biochemistry and Genetics, School of Medicine Lorestan University of Medical Sciences Khorramabad Iran; ^3^ Department of Microbiology, Faculty of Medical Sciences Islamic Azad University, Arak Branch Arak Iran; ^4^ Department of Midwifery Ministry of Health and Medical Education Tehran Iran; ^5^ Department of Epidemiology and Biostatistics, School of Health, Social Determinants of Health Research Center Birjand University of Medical Sciences Birjand Iran

**Keywords:** ATG16L1, ATG5, autophagy, HBV, HCC, polymorphism

## Abstract

**Background:**

Chronic hepatitis B (CHB) virus is the most common risk factor for developing liver malignancy. Autophagy is an essential element in human cell maintenance. Several studies have demonstrated that autophagy plays a vital role in liver cancer at different stages. In this systematic review, we intend to investigate the role of polymorphism and mutations of autophagy‐related genes (ATGs) in the pathogenesis and carcinogenesis of the hepatitis B virus (HBV).

**Materials and Methods:**

The search was conducted in online databases (Web of Science, PubMed, and Scopus) using Viruses, Infections, Polymorphism, Autophagy, and ATG. The study was conducted based on the Preferred Reporting Items for Systematic Reviews and Meta‐Analyses criteria.

**Results:**

The primary search results led to 422 studies. By screening and eligibility evaluation, only four studies were relevant. The most important polymorphisms in hepatocellular carcinoma were rs2241880 in ATG16L1, rs77859116, rs510432, and rs548234 in ATG5. Furthermore, some polymorphisms are associated with an increased risk of HBV infection including, rs2241880 in ATG16L1 and rs6568431 in ATG5.

**Conclusion:**

The current study highlights the importance of rs2241880 in ATG16L1 and rs77859116, rs510432, and rs548234 in ATG5 for HBV‐induced HCC. Additionally, some mutations in ATG16L1 and ATG5 were important in risk of HBV infection. The study highlights the gap of knowledge in the field of ATG polymorphisms in HBV infection and HBV‐induced HCC.

## 1 INTRODUCTION

Hepatitis B virus (HBV) is a member of the Hepadnaviridae family that comprises enveloped viruses with an icosahedral‐shaped capsid that harbors a partially double‐stranded, relaxed circular DNA (rcDNA) genome. There are four partially overlapping open reading frames (ORFs), named P (polymerase), S (surface), C (core), and X (HBx protein) in this ~3.2 kilobases genome.^1^ Phylogenetic analysis of obtained strains from all over the globe showed that there are 10 major genotypes (A–J) that vary in the whole genome length by >8%.^1^, ^2^ There are some differences in clinical outcomes and interferon treatment of patients based on the virus genotype; However, all of these genotypes are susceptible to the immunity produced by the vaccine.^2^, ^3^, ^4^

HBV is one of the most prevalent viral infections and a significant cause of liver disease worldwide.^5^ According to the World Health Organization, about one‐third of the world's population has been infected with HBV during their lifetime, especially during adulthood, which often results in a self‐limiting infection called acute hepatitis B.^1^, ^6^, ^7^, ^8^ This phase is primarily transient and asymptomatic; However, it can be severe by causing fatal fulminate which happens in about 0.5% of patients.^9^ The disease course can progress to the chronic hepatitis B (CHB) phase, in which stimulating inflammation triggers the fibrogenic process that eventually causes decompensated liver problems including, liver cirrhosis and hepatocellular carcinoma (HCC) and death happens in about one‐fourth of untreated patients.^1^ While most of the infections which acquired from mothers or during childhood transform into chronic; only fewer than 5% of immunocompetent adult patients face the chronic phase.^8^ Approximately 250 million people are living with CHB, of which one million lose their lives from the mentioned complications above every year. Currently, Africa, Asia, and some parts of Eastern and Central Europe are CHB‐endemic regions.^8^

The human cells are undergoing renovation and recycling to maintain homeostasis.^10^ To discard excess and damaged organelles, cells benefit from autophagy.^11^ Autophagy is a type of cell maintenance mechanism, in which cytoplasmic components are sequestrated by lysosomes. During autophagy, a double or multibound structure known as, autophagosome which is formed to degrade the cytoplasm. Afterward, the autophagosome fuses with the lysosome and creates, a new structure called autophagolysosome to remove the constituents.^11^, ^12^ Autophagy is a defense mechanism cells use to survive by recycling intracellular pathogens and nutrients.^11^, ^13^ On the other hand, if the apoptosis is inhibited, autophagy acts as a surrogate.^11^

Autophagy is known as a regulated process. The formation of autophagosomes depends on the transcription profile of a series of genes called autophagy‐related genes (ATGs).^14^ Factors that affect the expression of these genes can pose significant changes in the fate of the cells. Autophagy can impact cancer by different aspects including, the tumor microenvironment, cancer type, stage, and genetic background.^15^ Studies discovered mutations and polymorphism in ATGs in several human diseases including infections and cancers.^16^, ^17^

The HCC is known as one of the most common causes of cancer‐associated mortality.^18^ Conducted research represents that view, the malfunction in the autophagy in liver cells can lead to damage. This damage is due to a nonsufficient clearance for damaged organelles such as mitochondria. Furthermore, a specific form of autophagy known as xenophagy is critical for viral clearance from cells. The abnormal autophagy in cells could lead to countless damaged organelles, especially damage to mitochondria in the liver and reactive oxygen species (ROS) accumulation.^19^, ^20^ The autophagy in hepatocytes is a critical element due to the high turnover of energy. These high‐energy turnover, ROS accumulation, viral infection, and mitochondrial damage make autophagy an essential pathway for hepatocyte function. In this regard, autophagy‐defected liver cells represent fibrosis, cirrhosis, and HCC formation. On the other hand, HBV can affect the autophagy pathway by HBx protein during the infection.^20^, ^21^, ^22^, ^23^ By considering the critical role of autophagy in HCC generation and the precise role of HBV infection in tumorigenesis, we tried to evaluate host factors such as ATG gene polymorphisms' possible importance in HBV‐associated HCC. The current study aimed to investigate the role of polymorphism and mutations of ATGs in the pathogenesis and carcinogenesis of HBV.

## 2 MATERIALS AND METHODS

### 2.1 Search strategy and inclusion criteria

This study was designed based on the Preferred Reporting Items for Systematic Reviews and Meta‐Analyses criteria.^24^ The search was conducted in online databases including, Web of Science, PubMed, and Scopus, and by using Virus, Infections, Polymorphism, Autophagy, and ATG. The search query is provided in Supporting Information S1: File 1. The search was limited to November 21, 2023.

The inclusion criteria were considered English literature, original research, and relevant articles about evaluating ATG or other autophagy gene polymorphisms in viral infections without any date limitation. The time range for the search was from 1990 to November 21, 2023. Secondary studies or low‐eligible articles were excluded.

### 2.2 Data extraction

Three distinct authors reviewed and screened the search results, and extracted the included studies. The EndNote software (EndNote X9, Thomson Reuters) was used to generate a list of all of the studies and screening search results based on the inclusion criteria. For conflicts in the screening step, the third expert author strategy was used. First author name, year of publication, country, patient or control number, patient's age and gender, name, and prevalence of evaluated polymorphism and detection method were extracted from all included articles.

### 2.3 Risk of bias assessment

The Newcastle–Ottawa Quality Assessment Form was used to evaluate the risk of bias assessment and included studies' quality. The Newcastle–Ottawa Quality Assessment Form was used due to the study design of primary studies (case–control studies).^25^

## 3 RESULTS

### 3.1 Search results and quality assessment

**Figure 1:**
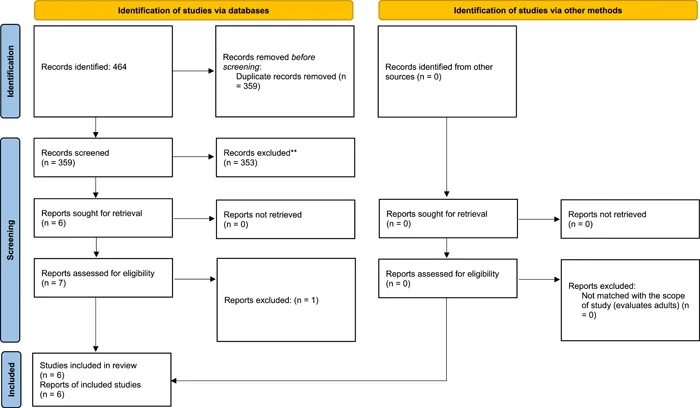
The study flowchart, search results, and eligibility.

The search results led to 464 studies. During the screening and eligibility, only four studies were relevant and used for data extraction. Figure 1 presents the flowchart of the study. The quality assessment for the included studies was provided in Supporting Information S1: Table 1. All included studies were evaluated for bias in Selection, Comparability, and Exposure, and the results were acceptable. In the search strategy, we tried to cover all viral infections and viral syndromes with autophagy ATG gene polymorphisms. In this regard, only five articles were included while four of the themes refer to HBV and HCC. The other study was excluded due to the heterogeneity of the evaluated virus.^26^

### 3.2 ATG polymorphisms in HBV patients

The evaluation of included studies leads to four different studies as listed in Table 1.^27^, ^28^, ^29^, ^30^ Current studies evaluated different polymorphisms such as rs2241880 in ATG16L1, rs6937876, rs548234, rs6568431, rs6937876, rs548234, rs6568431, rs6937876, rs548234, rs6568431, rs41292420, rs77859116, and rs510432 in ATG5 and rs2305686 in ATG7.

**Table 1:** Included studies for evaluation of ATG polymorphism in HBV infections and HBV‐associated HCC or cirrhosis.

Study features			Study sample				HBV detection method	Autophagy gene				Final conclusion	Ref.
First author name	Year	Country	Number		Age (years)	Gender (M/F)		Name	Polymorphism	Prevalence (%)	Detection method		
Wisetsathorn	2017	Thailand	CHB	114	49 ± 12	72/42	Serologic	ATG16L1	rs2241880 A > G	AG 54 (47.4) GG 6 (5.3) AA 54 (47.4)	PCR‐RFLP	An allele ATG16L1 rs2241880 was associated with an increased risk of HCC T allele ATG5 rs77859116 was significantly associated with the risk to HCC	[27]
								ATG5	rs41292420 C > T	TT 1 (0.9) TC 15 (13.2) CC 98 (86.0)			
									rs77859116 T > C	CC 0 CT 12 (10.5) TT 102 (89.5)			
									rs510432 T > C	TT 19 (16.0) TC 64 (56.1) CC 31 (27.9)			
								ATG7	rs2305686 C > A, T	TT 10 (8.8) TC52 (45.6) CC 52 (45.6)			
			HCC	102	56.8 ± 9.6	79/23		ATG16L1	rs2241880 A > G	AG 33 (32.4) GG 4 (3.9) AA 65 (63.7)			
								ATG5	rs41292420 C > T	TT 0 TC 17 (16.7) CC 85 (83.3)			
									rs77859116 T > C	CC 0 CT 0 TT 102 (100.0)			
									rs510432 T > C	TT 21 (20.6) TC 48 (47.1) CC 33 (32.4)			
								ATG7	rs2305686 C > A, T	TT 6 (5.9) TC 40 (39.2) CC 56 (54.9)			
			HC	131				ATG16L1	rs2241880 A > G	AG 65 (49.6) GG 11 (8.4) AA 55 (42.0)			
								ATG5	rs41292420 C > T	TT 1 (0.9) TC 14 (10.8) CC 116 (88.6)			
									rs77859116 T > C	CC 0 CT 0 TT 131 (100.0)			
									rs510432 T > C	TT 31 (23.7) TC 70 (53.4) CC 30 (22.9)			
								ATG7	rs2305686 C > A, T	TT 14 (10.7) TC 51 (38.9) CC 66 (50.4)			
Sharma	2020	Indian	HBV	551	38 ± 14	363/188	Serologic and PCR	ATG16L1	rs2241880 A > G	AA 125 (23) AG 266(48) GG 160(29)	PCR‐RFLP	rs2241880 mutant allele carriers were associated with an increased risk of hepatitis B virus infection	[28]
			CHB^a^	286	‐	‐				AA 55 (19.2) AG 153 (53.5) GG 78 (27.3)			
			C^a^	50	‐	‐				AA 11 (22) AG 22 (44) GG 17 (34)			
			HC	247	37 ± 18	118/129				AA 57 (23.1) AG 151(61) GG 39(15)			
Li	2019	China	HCC	113	40.37 ± 13.68	283/120	‐	ATG5	rs573775 T > C	CT 51 (45.1) TT 9 (8.0) CC 53 (46.9)	LDR‐PCR	These results indicate that rs510432 genotypes AA + GA are associated with disease progression and HCC risk in chronic HBV infection	[29]
									rs510432 G > A	GA 66 (58.4) AA 23 (20.4) GG 24 (21.2)			
			C	119					rs573775 T > C	CT 53 (44.6) TT 8 (6.7) CC 58 (48.7)			
									rs510432 G > A	GA 63 (52.9) AA 24 (20.2) GG 32 (26.9)			
			CHB	171					rs573775 T > C	CT 82 (47.9) TT 21 (12.3) CC 68 (39.8)			
									rs510432 G > A	GA 89 (52.1) GG 57 (33.3) AA 25 (14.6)			
			HC	196	38.58 ± 14.29	127/69			‐	‐			
Li	2020	China	CHB	171	‐	‐	Serologic	ATG5	rs6937876 G > A	GA or GG 106 (61.9) AA 65 (38)	LDR‐PCR	it is revealed for the first time that rs6568431 may be associated with susceptibility to HBV infection and that rs548234 may be associated with HCC risk in chronic HBV infection	[30]
									rs548234 C > T	TT 102 (59.6) TC or CC 69 (40.3)			
									rs6568431 A > C	CC 83 (48.5) CA or AA 89 (52)			
			HCC	113	48.93 ± 10.85	98/15			rs6937876 G > A	GA or GG 63 (55.7) AA 50 (44.2)			
									rs548234 C > T	TT 59 (52.2) TC or CC 54 (47.7)			
									rs6568431 A > C	CC 50 (44.2) CA or AA 63 (55.7)			
			C	119	‐	‐			rs6937876 G > A	GA or GG 50 (42) AA 69 (57.9)			
									rs548234 C > T	TT 82 (68.9) TC or CC 37 (31)			
									rs6568431 A > C	CC 66 (55.4) CA or AA 53 (44.5)			

Abbreviations: ATG, autophagy‐related gene; C, cirrhosis; CC, cervical cancer; CHB, chronic hepatitis B; HBV, hepatitis B virus; HC, healthy control; HCC, hepatocellular carcinoma; LDR‐PCR, ligase detection reactions‐polymerase chain reaction; P%, prevalence (%); PCR‐RFLP, polymerase chain reaction‐restriction fragment length polymorphism.

^a^ CHB and Cirrhosis are also included in the HBV patient population.

The most important polymorphisms in HCC were rs2241880 in ATG16L1, rs77859116, rs510432, and rs548234 in ATG5. Furthermore, some polymorphisms were associated with an increased risk of HBV infection including, rs2241880 in ATG16L1 and rs6568431 in ATG5. Studies were conducted between 2017 and 2020 and in Thailand, China, and India. Ligase detection reactions‐polymerase chain reaction and restriction fragment length polymorphism were used for the evaluation of mutations in HCC, Cirrhosis, CHB infection, and healthy controls.

## 4 DISCUSSION

The autophagic process protects cells from toxins, damaged proteins, and organelles.^31^ A wide range of diseases, including cancer, are associated with defects in the autophagy pathway.^32^ In cancer biology, autophagy is believed to play a dual role, suppressing tumors during the early stages of tumor development and promoting cell survival in established tumors. Therefore, autophagy has recently been considered a therapeutic target for various diseases, including cancer.^33^, ^34^ A complex genetic network consisting of multiple ATGs controls autophagy.^35^ Two ubiquitin‐like protein conjugation systems, termed ATG12 and ATG8 systems, are required for the formation of mammalian autophagosomes^36^ and ATGs (ATG3, ATG5, ATG7, ATG10, ATG12, ATG16L1, LC3) play a key role in this process.^37^ Desai et al. have recently shown that high expression levels of ATG7 are associated with poor patient survival in breast cancer.^38^ ATGs have also been implicated in developing other cancers.^39^, ^40^, ^41^ Several studies established the associations between ATGs and their polymorphisms in different types of cancers.^15^, ^42^ The homozygote deletion of ATG5 is associated with liver tumors in mouse models.^19^ It was also found that ATG5 point mutations are identified in gastric cancer, colorectal cancer, and HCC.^43^ In 2019, Shen and Lin^44^ evaluated the relationship between 14 variants of ATGs (ATG3, ATG5, ATG10, ATG12, ATG16L1) in HCC development. Among them ATG5 rs17067724, ATG10 rs1864183, ATG10 rs10514231, ATG12 rs26537, and ATG16L1 rs4663402 variants were notably associated with HCC. It seems that changes in the ATG gene's nucleotide sequence, affect the expression level of AGTs. This alteration in expression profile can alter autophagy flux in cells and it might explain the importance of host genetic and especially ATG polymorphisms in autophagy and HCC tumorigenesis during HBV infection.

PR domain zinc finger protein 1 (PRDM1), encoded by *prdm1* on chromosome 6q21.^45^ PRDM1 and ATG5 are both involved in the immunity and pathogenesis of chronic infections such as CHB and cancers.^46^, ^47^ In 2019, Li et al. revealed for the first time that the genetic variants in the PRDM1‐ATG5 region are associated with CHB infection. Through this study, they identified that rs6568431 may influence susceptibility to HBV infection, while rs548234 may influence the risk of HCC.^30^ Wisetsathorn et al. investigated the association between ATG5 and ATG16L1 variants and susceptibility to CHB infection with HCC development. The study results indicate that A allele of ATG16L1 rs2241880 (T300A) increases the risk of developing HCC compared to CHB patients without HCC and healthy controls. The t allele of ATG5 rs77859116 has notably increased susceptibility to the HCC in comparison to CHB patients without HCC.^27^ These findings suggested the crucial role of the autophagy genes in the risk of HCC development and HBV infection susceptibility and the ATG genes at the center of tumorigenesis events. The ATG16L1 is a key gene involved in autophagosome formation and modulating inflammation,^48^ which can lead to gastric adenocarcinoma. Burada et al. have found that ATG16L1 rs2241880 polymorphism may affect gastric cancer susceptibility and the G allele plays a protective role against gastric cancer.^35^ According to reports, autophagy may induce tumor progression by facilitating metastasis.^49^, ^50^ On the other hand, in 2017, a study showed that ATG16L1 rs2241880 was linked to a decrease in metastasis in patients with colorectal cancer.^51^ In 2021, Jamali et al. evaluated the association of ATG16L1 rs2241880 polymorphism with colorectal cancer risk in an Iranian population and revealed that ATG16L1 rs2241880 increases the risk of colorectal cancer.^52^ Bueno‐Martínez et al., could not find any association between ATG16L1 rs2241880 and the susceptibility to glioblastoma cancer.^53^ An association of ATG16L1 rs2241880 polymorphism with HCC also has been observed in the Reuken et al. study. They found a higher prevalence of G allele of ATG16L1 rs2241880 in patients with HCC compared to controls without HCC.^54^ The results of the current study represent that the most important polymorphisms in HCC patients in comparison with CHB patients were ATG16L1 rs2241880 and rs77859116, rs510432, and rs548234 in ATG5. Furthermore, rs2241880 in ATG16L1 and rs6568431 in ATG5 polymorphisms were associated with an increased risk of HBV infection. A review of these findings could lead us to the critical role of autophagy in tumorigenesis and the effects of the ATG polymorphisms, especially ATG16L1, in cancer development and metastasis. In favor of this information, some therapeutic approaches are suggested for autophagy alteration. As a general point, during the HCC treatment by chemotherapy an increase in autophagy is the essential element, that could be affected by chemotherapy agents.^55^

As we mentioned earlier, some of the mutations including rs2241880 in ATG16L1 and rs77859116, rs510432, and rs548234 in ATG5 seem to be associated with HBV‐induced HCC. In this regard, another aspect is about the molecular mechanisms behind these mutations and their pathophysiological effects. The ATG16L1 is an essential element for the formation of a double membrane‐shaped autophagosome. The rs2241880 (Thr300Ala) is located in coding exon 9 of ATG16L1.^56^, ^57^ The ATG16 also is associated with ATG5 and ATG12 for ATG16 final complex formation. This function makes the ATG16L1 an interesting gene during autophagy.^58^ The rs2241880 makes ATG16L1 more susceptible to caspase‐3 mediated degradation that leads to autophagy alteration.^59^ This polymorphism can also affect inflammatory pathways. The rs2241880 can increase the interleukin (IL)‐1β and IL‐6 expression which leads to augmentation of inflammation.^60^ These inflammation and elevated levels of IL‐6 could be considered as a start for oncogenesis and oncogenic mutations due to the IL‐6–STAT3 axis.^61^

The ATG5 is a critical element for autophagosome formation by interaction with ATG16 and ATG12. A rs77859116 in ATG5 can interfere with ATG5 binding to ATG12 and ATG16. Also, rs77859116 leads to destabilizing ATG5. Both of these interferences can lead to decreased autophagy and might be a clue for the carcinogenesis of this polymorphism.^62^ The rs510432 is a polymorphism upstream of ATG5.^63^ The rs510432 affects the ATG5 expression levels and decreases its transcription.^42^ The transcription of ATG5 by rs510432 is due to the reduced affinity of metal‐responsive transcription factor 1 attachment into the mutant ATG5 promoter.^64^ This downregulated ATG5 might be a reason for the possible oncogenesis of rs510432. The same mechanism for rs548234 in ATG5 is suggested for its possible role in autophagy alteration.^65^

All these point us to the necessity of future investigations in the field of autophagy and HCC or even other malignancies for the possibility of clinical applications such as therapeutic and even diagnostic or prognostic. In addition, all evaluated primary studies in our current study were from India, Thailand, and China. There were no reports from other countries. The fact of various patterns of polymorphism distribution in different ethnicities in HCC patients^66^, ^67^ highlights the critical importance of future studies for ATG polymorphism in HCC development in other countries.

It should be noted that our current study has some limitations. A significant limitation of this study is the limited number of primary studies. This limitation also highlights the importance of further primary original studies in this field of study. Another limitation is limited data about other types of malignancies and infections regardless of HCC and HBV. In the search strategy, we tried to include all viral infections and autophagy polymorphisms, which all of the conducted studies were related to HBV and HCC. In this regard, we suggested a narrower search strategy for further studies.

In conclusion, the current study highlights the importance of rs2241880 in ATG16L1 and rs77859116, rs510432, and rs548234 in ATG5 for HBV‐induced HCC. Furthermore, some mutations in ATG16L1 and ATG5 were more prevalent in HBV‐infected patients in comparison with healthy controls, which seems to be important in the risk of HBV infection.

## AUTHOR CONTRIBUTIONS

Parastoo Yousefi, Hamid Salehiniya, and Alireza Tabibzadeh designed and conceived the study. Parastoo Yousefi, Abdulhussain Kadhim Jawaziri, and Hamid Salehiniya collected the data. Mohsen Mehrjoo, Mandana Akhavan, Leila Allahqoli, and Parastoo Yousefi extracted and interpreted the data. All authors drafted the manuscript. Hamid Salehiniya and Parastoo Yousefi provided administrative, technical, or material support. Leila Allahqoli and Alireza Tabibzadeh provided oversight. All authors contributed to the article and approved the submitted version.

## CONFLICT OF INTEREST STATEMENT

The authors declare no conflict of interest.

## Supporting information

Supporting information.
