# Supplementary material for: Autophagy‐related genes polymorphism in hepatitis B virus‐associated hepatocellular carcinoma: A systematic review
Source: Immun Inflamm Dis. 2024 Feb 14;12(2):e1182. doi: 10.1002/iid3.1182 (PMC10865419; doi:10.1002/iid3.1182)
Supplement: Supplementary file 1 — Supporting information. [file IID3-12-e1182-s001.docx]

**Supplement files**

Supplement file 1:

Search query

((("Virus Diseases"[Mesh]) OR ((((((((Virus Disease[Title/Abstract]) ) OR (Virus Infections[Title/Abstract])) OR (Virus Infection[Title/Abstract])) OR (Viral Diseases[Title/Abstract])) OR (Viral Disease[Title/Abstract])) OR (Viral Infections[Title/Abstract])) OR (Viral Infection[Title/Abstract]))) AND (("Polymorphism, Genetic"[Mesh]) OR ((((((((Polymorphisms, Genetic[Title/Abstract]) OR (Genetic Polymorphism[Title/Abstract])) OR (Genetic Polymorphisms[Title/Abstract])) OR (Gene Polymorphism[Title/Abstract])) OR (Gene Polymorphisms[Title/Abstract])) OR (Polymorphism, Gene[Title/Abstract])) OR (Polymorphism[Title/Abstract])) OR (Polymorphisms[Title/Abstract])))) AND (("Autophagy"[Mesh]) OR (((((((((Autophagy, Cellular[Title/Abstract]) OR (Cellular Autophagy[Title/Abstract])) OR (Autophagocytosis[Title/Abstract])) OR (Reticulophagy[Title/Abstract])) OR (ER-Phagy[Title/Abstract])) OR (Nucleophagy[Title/Abstract])) OR (Ribophagy[Title/Abstract])) OR (Lipophagy[Title/Abstract])) OR (ATG[Title/Abstract])))

Supplement table 1:

Studies quality assessment

| Study features | | | Quality assessment | | | Ref |
| --- | --- | --- | --- | --- | --- | --- |
| Frist author name | Year | Country | Selection | Comparability | Exposure |  |
| Wisetsathorn | 2017 | Thailand | **** | ** | *** | (1) |
| Sharma | 2020 | Indian | **** | * | *** | (2) |
| Li | 2019 | China | **** | * | *** | (3) |
| Li | 2019 | China | **** | * | *** | (4) |

1. Wisetsathorn S, Tantithavorn V, Hirankarn N, Tangkijvanich P, Saethang T, Kimkong I. Gene polymorphisms of autophagy machinery and the risk of hepatitis B virus-related hepatocellular carcinoma in a Thai population. Scienceasia. 2017;43:362-8.

2. Sharma A, Kaur S, Duseja A, Changotra H. The autophagy gene ATG16L1 (T300A) variant is associated with the risk and progression of HBV infection. Infection, Genetics and Evolution. 2020;84:104404.

3. Li N, Fan X, Wang X, Deng H, Zhang K, Zhang X, et al. Autophagy-related 5 gene rs510432 polymorphism is associated with hepatocellular carcinoma in patients with chronic hepatitis B virus infection. Immunological investigations. 2019;48(4):378-91.

4. Li N, Fan X, Wang X, Zhang X, Zhang K, Han Q, et al. Genetic association of polymorphisms at the intergenic region between PRDM1 and ATG5 with hepatitis B virus infection in Han Chinese patients. Journal of medical virology. 2020;92(8):1198-205.
